# Lyme Disease Presenting With Interesting Neurological Features of Weakness and Hyporeflexia: A Case Report

**DOI:** 10.7759/cureus.43296

**Published:** 2023-08-10

**Authors:** Mehak Semy, Peterkin Lee-kwen, Sarfaraz Semy

**Affiliations:** 1 Internal Medicine, Dr. Dnyandeo Yashwantrao (DY) Patil School of Medicine, Mumbai, IND; 2 Neurology, Mercy Hospital of Buffalo, Buffalo, USA

**Keywords:** atypical infection, neuro-critical care, neurology and critical care, lyme disease and other tick borne pathogens, tick-borne infections, atypical presentation, an unusal case, lyme neuroborreliosis, neurological lyme, lyme's disease

## Abstract

Lyme disease is a tick-borne bacterial infection caused primarily by three pathogenic species of spirochete *Borrelia* (*B. burgdorferi*, *B. afzelii*, and *B. garinii*). It has a wide range of clinical manifestations ranging in severity. Although, it is generally divided into three phases: early localized, early disseminated, and late disease. Certain cases do not follow the same order described in standard books like Harrison's. Thus, it is vital to establish a chronological timeline when establishing the diagnosis. Here, we describe a 25-year-old female with numbness and tingling that began in her torso and then spread to her entire body. Physical examination revealed diminished motor reflexes and power, but the diagnosis of neuroborreliosis with monoradiculitis was only established with positive laboratory antibody evaluation and lumbar puncture. The patient’s symptoms resolved quickly with a four-day inpatient course of IV ceftriaxone followed by 10 days of oral doxycycline.

## Introduction

Lyme disease is a spirochaetal infection transmitted by Ixodes ticks. It was first described in the year 1977 as "Lyme arthritis" in children in Connecticut who were initially given a diagnosis of juvenile rheumatoid arthritis [1]. It typically presents in woodland areas with a temperate climate. Early manifestations include fever, headache, fatigue, and a target skin-like rash called erythema migrans. If untreated it can disseminate to the joints, heart, and nervous system. Besides the skin, the nervous system is Lyme's second most affected system [2]. The spirochetes can spread to the nervous system during early disseminated disease, often leading to a triad of lymphocytic meningitis, cranial neuritis most commonly affecting the facial nerve, and radiculoneuritis [3]. These clinical manifestations can occur alone or in combination.

## Case presentation

A 25-year-old right-handed female with moderate, well-controlled asthma presented to the neurology outpatient clinic with complaints of progressive numbness and tingling which started four weeks ago. It started on the right side of her stomach where she had her cesarean section and radiated to her back and then to her entire body. The tingling was not associated with any burning or pins and needle sensation. The patient mentioned that she did not pay much attention to it initially, but over the next two days, her symptoms intensified which led her to visit her primary care doctor who, due to her former history of asthma, thought that she was just having an anxious reaction to it. She was prescribed methylprednisolone and an albuterol inhaler but noticed no improvement. She especially became alarmed when she could not hold her toddler anymore and ended up dropping the child secondary to her numbness and tingling. The patient did not have a history of recent fever, chills, headache, sore throat, or rash. She did not have any notable history of travel or insect bite.

The patient was conscious and oriented to time, place, and person on examination. Her vitals were unremarkable with a temperature of 98.6 degrees Fahrenheit, heart rate of 83 beats/min, and blood pressure of 120/82 mmHg. Motor examination revealed decreased tone more significant in the upper limbs (3/5) than in the lower limbs (4/5) along with hyporeflexia in the biceps, triceps, patellar, and Achilles. She had a normal sense of proprioception and vibration along with a negative Babinski sign. Due to the progressive weakness of her bilateral upper extremities, she was admitted to the hospital and her initial workup included a complete blood count (Table 1), basic metabolic profile (Table 2), magnesium (1.9 mg/dl; normal: 1.7-2.5 mg/dl), phosphorus (3.1 mg/dl; normal: 2.5-5.0 mg/dl), vitamin B12 levels (244 pg/ml; normal: 180-914 pg/dl), ganglioside GM-1 antibodies (negative), creatine kinase isoenzymes (50 U/L; normal: 30-135 U/L), c-reactive protein (0.5 mg/dl; normal: 76.2 nmol/L), erythrocyte sedimentation rate, and Lyme disease antibody testing. The laboratory analysis revealed everything to be in a mildly elevated erythrocyte sedimentation rate (17 mm/hr; normal: 0-10 mm/hr) and a positive antibody screening for Lyme disease.

**Table 1:** Complete blood count MCV: mean corpuscular volume, MCH: mean corpuscular hemoglobin. It shows all the tested values within the reference range.

Test	Values	Reference range
Leukocytes (10*3/uL)	6.2	4.0-11.0
Erythrocytes (10*6/uL)	4.40	4.1-5.6
Hemoglobin (g/dl)	13.0	12.0-16.0
Hematocrit (%)	37.7	36.0-47.0
Erythrocyte MCV (fL)	85.6	81.0-99.0
Erythrocyte MCH (pg)	29.5	26.0-34.0
Erythrocyte mean corpuscular hemoglobin concentration (g/dl)	34.4	31.0-37.0
Erythrocyte distribution width (%)	13.6	11.5-14.5
Platelets (10*3/uL)	268	145-450
Platelet mean volume (fL)	8.7	7.4-10.4
Neutrophils/100 leukocytes (%)	68	50-75
Lymphocytes/100 leukocytes (%)	20	20-40
Monocytes/100 leukocytes (%)	7	2-10
Eosinophils/100 leukocytes (%)	4	0-8
Basophils/100 leukocytes (%)	1	0-2
Neutrophils (10*3/uL)	4.2	2.0-8.2
Lymphocytes (10*3/uL)	1.2	0.8-4.4
Monocytes (10*3/uL)	0.4	0.1-1.1
Basophils (10*3/uL)	0.2	0.0-0.6

**Table 2:** Basic metabolic profile GFR: glomerular filtration rate. It shows all the tested values within the reference range.

Test	Value	Reference
Glucose (mg/dl)	84	74-100
Urea nitrogen (mg/dl)	12	7-25
Creatinine (mg/dl)	0.7	0.6-1.10
Urea nitrogen/creatinine	17.1	10.0-20.1
Sodium (mmol/L)	138	136-145
Potassium (mmol/L)	3.8	3.6-5.1
Chloride (mmol/L)	107	98-107
Carbon dioxide (mmol/L)	21	21-31
Anion gap	9	3-11
Calcium (mg/dl)	8.6	8.6-10.3
GFR/1.73 sqm (mL/min/1.73 m2)	>90.0	>=90.0

Western blot for Lyme confirmed two of three positive immunoglobin M (IgM) and one of 10 immunoglobin G (IgG). This confirmed an early stage of infection (Table 3). An upper limb electromyography evaluation demonstrated reduced recruitment during voluntary contraction and early fatigue during a sustained muscle contraction. MRI of the brain was ordered and was negative for meningeal enhancement. A lumbar puncture revealed elevated protein concentration (148 mg/dl; normal: 15-60 mg/dl) and normal glucose count (45 mg/dl; normal: 50-80 mg/dl).

**Table 3:** Western blot test for Lyme's disease antibody levels showing IgM positive (2/3) significant with early Lyme's disease IgG: immunoglobulin G, IgM: immunoglobulin M. As per the Centers for Disease Control and Prevention, the criteria for positive Western blot analysis for serologic confirmation of Lyme disease IgM immunoblot must show reactivity to two out of three *Borrelial* protein bands to be considered positive. Similarly, IgG immunoblot must show reactivity to five out of ten *Borrelial* protein bands to be considered positive.

Name	Value	Reference range
Lyme disease antibody (IgM), blot	Positive	Negative
24-KD (IgM) band	Reactive	
39-KD (IgM) band	Reactive	
41-KD (IgM) band	Non-reactive	
Lyme disease antibody (IgG), blot	Negative	Negative
18-KD (IgG) band	Reactive	
23-KD (IgG) band	Non-reactive	
28-KD (IgG) band	Non-reactive	
30-KD (IgG) band	Non-reactive	
39-KD (IgG) band	Non-reactive	
41-KD (IgG) band	Non-reactive	
45-KD (IgG) band	Non-reactive	
58-KD (IgG) band	Non-reactive	
66-KD (IgG) band	Non-reactive	
93-KD (IgG) band	Non-reactive	

She was started on IV ceftriaxone 2 g once daily. Three days after the treatment initiation, the patient was transitioned to oral doxycycline, a dose of 100 mg twice daily for the next 10 days, and discharged home. At the time of discharge, the patient was able to move all extremities spontaneously and ambulate with a normal gait. She was instructed to follow up with a neurology outpatient to check on her progress a week after discharge.

Upon following up with the outpatient clinic, they informed us that the patient has been recovering well with only minimal residuary weakness. They will continue to follow her up in case anything changes.

## Discussion

The rapid rise in Lyme disease also appears to be due to environmental factors like climate change which is facilitating Ixodes tick survival rate making it the most reported tick-borne disease in the United States [4-5]. Although this case was in a Lyme endemic zone, not all cases can be captured through routine surveillance. Since the disease is transmitted only by Ixodes ticks, the possibility of exposure remains high, but not everybody who does end up with Lyme’s remembers the instance of exposure.

Approximately one-quarter of all Lyme disease-associated cranial neuropathies involve the facial nerve [6]. The involvement of the T10-T12 level which primarily provides innervation to the periumbilical area, hypogastric region, and middle back in this patient is a rare phenomenon and not characteristic of Lyme disease. Older European studies suggested that this could be associated with the tick bite site, but this issue has not been addressed in detail [7].

Traditionally, two-tier testing is performed, starting with a whole cell-based enzyme-link immunosorbent assay (ELISA), to estimate and measure the total antibodies against the causative organism followed by a more specific serologic test, the Western blot with different IgM and IgG blots [6]. The immunoassay is used to rule out the disease since Western blot testing can be difficult to perform and interpret. Serologic testing alone can neither exclude nor establish the diagnosis of Lyme disease; thus, clinical manifestations are essential to substantiate the diagnosis.

The involvement of the nervous system can be segregated into three potentially intersecting categories, including parenchymal, like meningitis, extra parenchymal, like memory impairment, confusion, or unifocal or multifocal inflammation of the peripheral nerves [8-9]. In a patient with symptoms involving the peripheral nerves, Lyme disease should be suspected especially in endemic areas in the months of June and July. The patients normally do not have any apparent symptoms just like this patient whose only symptom was tingling with hyporeflexia.

Thus, further testing is always appropriate to exclude any other differential diagnoses like Guillain-Barré syndrome (GBS), metabolic and electrolyte abnormalities (hypophosphatemia), compressive myelopathy, and post-herpetic neuralgia. Our patient showed progressive hyporeflexia, loss of muscle tone, and high cerebrospinal fluid protein which puts GBS also as one of our top differentials. However, the flaccid paralysis in the patient was more in the upper limbs than the lower limbs, and this is not a feature of pathognomonic to GBS [10]. Hence, the clinical manifestations, the positive Lyme antibody screening, and Western Blot testing as well as the rapid response rate to the antibiotics helped establish Lyme disease to be the primary source of the symptoms. Neuroimaging, especially MRI, electromyography, and lumbar puncture is also helpful to confirm the diagnosis [11].

The health of the muscle and the nerve cells controlling them is monitored using electromyography. Early fatigue reveals the premature tiring of the muscle either due to a decreased action potential or less frequent contraction. The weakness due to reduced motor recruitment demonstrates the inability of the nervous system to engage an adequate number of motor units to produce a strong muscle contraction. This can occur in Lyme disease as it leads to inflammation which can damage the nerves affecting their ability to transmit signals to the muscles resulting in weakness.

The presence of inflammatory cerebrospinal fluid fortifies the diagnosis, but it does not alter the treatment measures since the drugs used for management already provide moderately good cerebrospinal fluid penetration.

The treatment regimens for patients with acute neurologic features are based on randomized and prospective open-label trials conducted in European countries [12-14] as well as small case series from the United States. It typically consists of either oral doxycycline (100 mg once/twice daily) or IV ceftriaxone (2 g once daily) or IV cefotaxime (2 g every eight hours) for 14-21 days. Doxycycline has a bioavailability of more than 98% and moderately good penetration into the cerebrospinal fluid making its oral dosage equal to that of IV drugs [14-15]. IV ceftriaxone and IV cefotaxime are both equally efficacious as doxycycline in treating patients [13]. However, most patients with neurologic Lyme disease still receive oral doxycycline, and IV therapy is preferred in patients who require hospitalization and cannot take doxycycline orally with a transition to oral doxycycline upon discharge.

## Conclusions

Early disseminate Lyme neuroborreliosis is rare and usually occurs several weeks after a tick bite. This case of Lyme disease did not present itself with the common symptoms like arthralgia, fever, rash, or regional lymphadenopathy but in fact exhibited decreased reflexes, numbness, and tingling particularly near the hypogastric region making it distinct from the classical presentation. To rule out all the other differentials and confirm the diagnosis of Lyme disease in this kind of presentation, a high degree of awareness is required. Physicians need to be aware of the non-pathognomonic timeline of the disease at presentation since the clinical presentation of the disease can differ significantly from one individual to another. Early diagnosis and antibiotic treatment are also of prime importance to prevent any long-standing complications as well as aid the patient’s anxiety.
